# Optimizing early enteral nutrition in severe stroke (OPENS): protocol for a multicentre randomized controlled trial

**DOI:** 10.1186/s12883-019-1253-2

**Published:** 2019-02-12

**Authors:** Fang Yuan, Fang Yang, Wei Zhang, Yi Jia, Yaling Ma, Yongcai Qu, Xinglai Wang, Kang Huo, Chengkai Wang, Xiangjun Yuan, Chaohui Song, Bei Zhang, Wen Jiang, on behalf of the OPENS study group

**Affiliations:** 1 Department of Neurology, Xijing Hospital, Fourth Military Medical University, Xi’an, 710032 China; 2 Department of Neurology, Tangdu Hospital, Fourth Military Medical University, Xi’an, 710038 China; 3 Department of Neurology, Xi’an Gaoxing Hospital, Xi’an, 710075 China; 4 Department of Neurology, Yulin No.1 Hospital, Yulin, 719000 China; 5 Department of Neurology, Yan’an University Affiliated Hospital, Yanan, 716000 China; 6 Department of Neurology, Xi’an Central Hospital, Xi’an, 710003 China; 7 Department of Neurology, The First Affiliated Hospital of Xi’an Jiaotong University, Xi’an, 710061 China; 8 Department of Neurology, Tongchuan People’s Hospital, Tongchuan, 727000 China; 9 Department of Neurology, Weinan Central Hospital, Weinan, 714000 China; 10 Department of Neurology, Tongchuan Mining Bureau Hospital, Tongchuan, 727000 China; 11 Department of Neurology, The First Affiliated Hospital of Xi’an Medical University, Xi’an, 710077 China; 12 The Shaanxi Cerebrovascular Disease Clinical Research Center, Xi’an, 710032 China

**Keywords:** Enteral nutrition, Severe stroke, Permissive underfeeding, Prokinetic drug, Critical care, Protocol

## Abstract

**Background:**

Malnutrition is one of the crucial factors associated with poor prognosis in critical ill patients, yet a significant evidence gap surrounds the management of initial enteral feeding in severe stroke. The Optimizing Early Enteral Nutrition in Severe Stroke (OPENS) trial will compare a strategy of modified full enteral nutrition (EN) (standard full EN in conjunction with prokinetic drug) and a strategy of permissive underfeeding (40 to 60% of estimated caloric requirements) with standard full EN (advancement to target nutrition goals) in patients with severe stroke.

**Methods:**

The OPENS trial is a multicenter randomized controlled study. A total of 600 adult patients with severe stroke will be enrolled in 12 study sites in China, and randomized to standard full EN, modified full EN, or permissive underfeeding. The primary outcome measurement is the proportion of participants with a poor outcome (modified Rankin Scale ≥3) at day 90 of enrollment. Secondary outcomes include incidence rates of complications during hospitalization, disability at hospital discharge, and the ability of activities of daily living at day 90 of enrollment. The relationship between intervention and the primary outcome will be analyzed using multivariate logistic regression adjusted for study site, demographics, and baseline characteristics.

**Discussion:**

The OPENS trial will explore the optimum initial feeding strategy for acute severe stroke. This trial is, therefore, an important step in bridging the evidence gap surrounding the enteral feeding for patients with severe stroke during the first week of hospitalization.

**Trial registration:**

ClinicalTrials.gov Identifier: NCT02982668; First Posted: December 5, 2016.

## Background

Despite advances in prevention and treatment over the past century, stroke remains the third major cause of death worldwide [1, 2] and the leading cause of mortality and disability in China [3]. As a previously underestimated problem, malnourishment is one of the most common complications of acute stroke and is strongly associated with an increased prevalence of impaired immunologic function, a high mortality rate, and unfavorable outcomes in long term [4–7]. Almost half of the stroke patients have dysphagia, which impedes the oral intake of nutrition and deteriorates the nutritional status [8, 9]. Nutrition support is particularly critical in patients with severe stroke due to the high prevalence of consciousness impairment and intense stress responses which lead to inability of oral nutrition, altered gastrointestinal motility, and intestinal barrier dysfunction [10].

It has been widely accepted that early enteral nutrition (EN) reduces case fatality in dysphagic stroke patients compared with withholding or delaying enteral tube feeding [11, 12]. EN maintains the functional integrity of the gut and prevents increased gut permeability, thus it reduces the likelihood of systemic infection and higher risk for multiple-organ dysfunction syndrome [13, 14]. However, the appropriate dose of daily caloric intake for critically ill patients in the first week of hospitalization lacks a clear consensus. Initial trophic EN (up to 500 kcal/d) was shown to have similar outcomes with full EN (advancement to target nutrition goals) in patients with acute respiratory injury, and it resulted in a lower incidence of gastrointestinal intolerance [15, 16]. In another study with 894 critically ill patients, permissive underfeeding (40 to 60% of estimated caloric requirements) combined with full protein provision also had similar outcomes as standard feeding, even among patients with high nutritional risk [17, 18]. However, the above studies did not target at patients with severe stroke who have distinct clinical characteristics, and there is an evidence gap surrounding the enteral feeding for them during the first week of hospitalization. Moreover, previous studies showed that adding prokinetic drugs could reduce gastric residual volumes and improved tolerance of EN in critically ill patients [19–21], but further studies are needed to investigate whether the use of prokinetic would reduce the risk of aspiration and mortality in patients with severe stroke.

In this multicenter, randomized, controlled study, we proposed two different strategies of EN for patients with severe stroke, one was in the perspective of reducing energy provision (permissive underfeeding) and the other was in the light of increasing gastrointestinal motility (full EN in conjunction with prokinetic drug), and compared them with a strategy of standard full EN in functional outcomes and incidence rates of complications.

## Methods

### Study objectives

The objectives of OPENS study are: (1) to assess the effects of standard full EN, modified full EN (standard full EN in conjunction with prokinetic drug), and permissive underfeeding on the three-month mortality and morbidity of patients with severe stroke; (2) to test whether modified full EN or permissive underfeeding would result in a lower incidence of gastrointestinal intolerance and nosocomial infections in patients with severe stroke compared with standard full EN.

### Study design

**Fig. 1:**
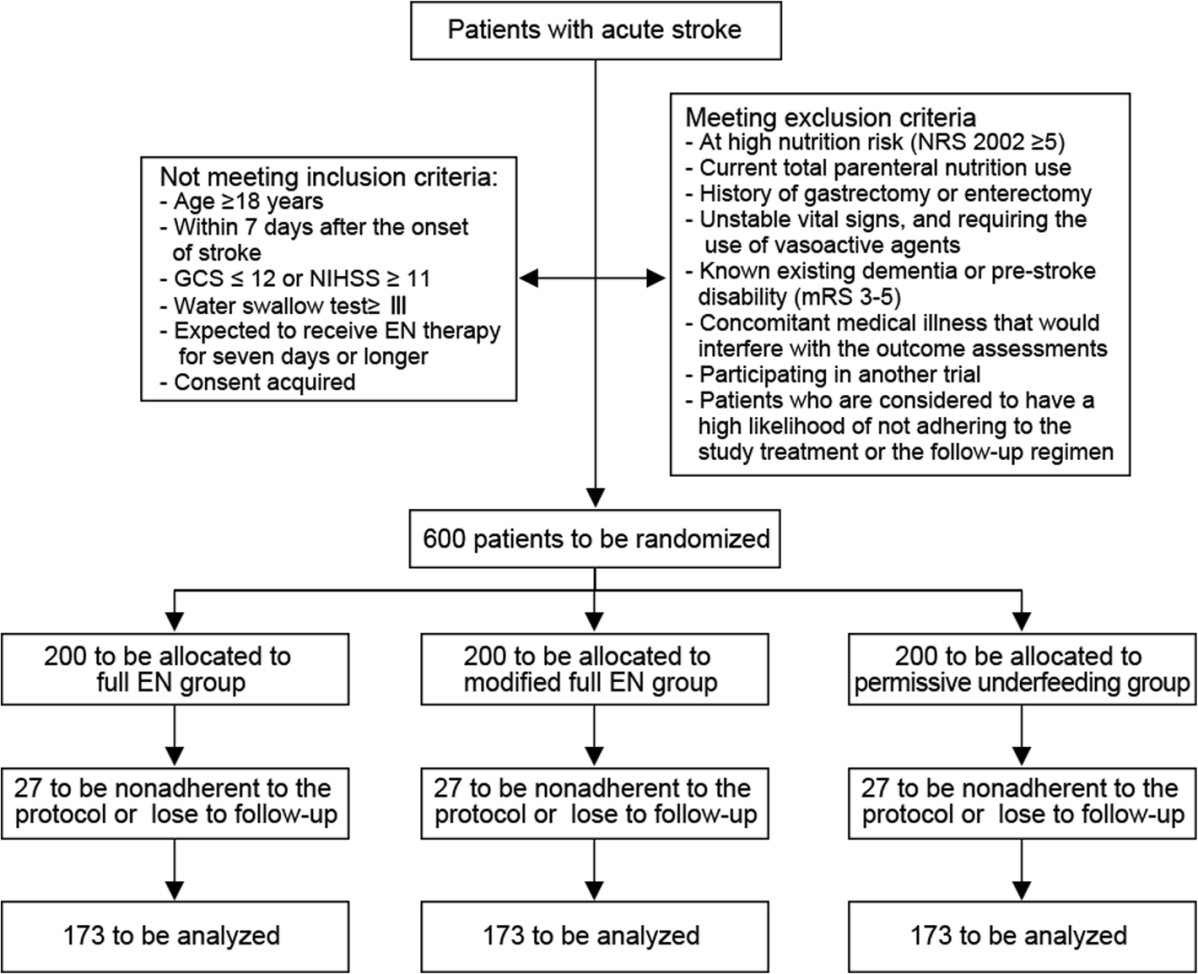
Flow chart of participants in the OPENS trial. EN, enteral nutrition; GCS, Glasgow Coma Scale; mRS, modified Rankin Scale; NIHSS, National Institute of Health stroke scale; NRS 2002, Nutritional risk screening 2002

This study has a prospective, multicenter, randomized, controlled design. Patients will be randomized to three different arms: (1) standard full EN group, provided with 70–100% of caloric requirements; (2) modified full EN group, provided with 70–100% of caloric requirements and administered with prokinetic agent in preventive use; (3) permissive underfeeding group, provided with 40–60% of caloric requirements. All the patients are blind to the grouping results. The grouping results are open for the researchers since they have to follow a prescribed strategy of EN provision. Figure 1 shows a flow diagram of the study design. This multicenter trial involves 12 tertiary and district general hospitals in Shaanxi province, an administrative region with a population of 38 million in northwest China. The study will be conducted according to Good Clinical Practice guidelines and the Declaration of Helsinki. The protocol has been approved by the ethics committees of Xijing hospital (KY20162086–2).

### Training of investigators

All the investigators participating in the OPENS trial have to receive trainings on the Good Clinical Practice, study protocol, and use of Glasgow Coma Scale (GCS), National Institute of Health stroke scale (NIHSS), Nutritional risk screening (NRS 2002), Barthel Index, and modified Rankin Scale (mRS).

### Study population

The study will recruit patients with acute severe stroke who are unable to maintain volitional intake. In order to be eligible for inclusion in the study the patients.

have to comply with the following criteria: (1) Age ≥ 18 years; (2) The randomly.

assigned EN regimen is able to be commenced within 7 days after the onset of stroke (ischaemic or haemorrhagic), confirmed by a CT or MRI scan of the brain (If the precise timing of the onset of symptoms or signs of the qualifying event is unknown, then the time of onset will be taken as the last time the patient was known to be well.); (3) GCS on admission ≤12 or NIHSS on admission ≥11; (4) Water swallow test≥ III; (5) Expected to receive EN therapy for 7 days or longer; (6) Written informed consent is able to be obtained directly from the patient or an appropriate surrogate, based on local ethics committee recommendations.

Exclusion criteria are: (1) At high nutrition risk (NRS 2002 ≥ 5); (2) Current total parenteral nutrition (TPN) use; (3) History of gastrectomy or enterectomy; (4) Unstable vital signs, and requiring the use of vasoactive agents; (5) Known existing dementia or pre-stroke disability (e.g. mRS 3–5); (6) Concomitant medical illness that would interfere with the outcome assessments and/or follow-up (a. Advanced cancer; b. Severe pulmonary dysfunction [Forced expiratory volume in 1 second < 50%]; c. Severe cardiac dysfunction [Ejection fraction ≤50%]; d. Severe hepatic failure [Child-Pugh score ≥ 7]; e. Severe renal failure [Glomerular filtration rate ≤ 30 mL/min or serum creatinine ≥4 mg/dL]); (7) Currently participating in other investigational trials; (8) Patients who are considered to have a high likelihood of not adhering to the study treatment or the follow-up regimen.

### Randomization

The centralized randomization (computerized random numbers) is performed in a secure website (http://traillogin.applinzi.com). Participants will be randomized into one of the three intervention arms (1:1:1).

### The enteral nutrition protocol

#### Intervention arms

For patients in standard full EN group, the target on Day 1 is one third of the estimated need, the target on Day 2 is half of the estimated need, and the targets from Day 3 to Day 7 are 100% of the estimated need with the acceptable range being 70–100%. For patients in modified full EN group, the caloric targets from Day 1 to Day 7 are the same with standard full EN group, but they will be administered with prokinetic drug (metoclopramide doses of 10 mg 4 times a day or erythromycin doses of 3–7 mg/kg/d) from Day 1 to Day 7. For patients in permissive underfeeding group, the target on Day 1 is one third of the estimated need, and the targets from Day 2 to Day 7 are 60% of the estimated need with the acceptable range being 40–60%. After Day 7, the caloric target for all the participants is 100% of the estimated need. The amount of protein provided for all the participants since Day 1 are the same: 1.2–1.5 g/kg /d. Table 1 presented the enteral feeding regimens in these three groups.

**Table 1:** Enteral feeding regimens

	Standard Full EN	Modified Full EN	Permissive Underfeeding
Estimated caloric need	BMI 18–30: daily caloric requirement (kcal) = actual weight (in kilos) × 25 (kcal) BMI < 18 or > 30: daily caloric requirement (kcal) = [height (in cm) – 105] × 25 (kcal)		
Caloric provision	Day 1: 1/3 of the estimated need Day 2: 1/2 of the estimated need Day 3–7: 100% of the estimated need with the acceptable range being 70–100%. After day 7: 100% of the estimated need	Day 1: 1/3 of the estimated need Day 2: 1/2 of the estimated need Day 3–7: 100% of the estimated need with the acceptable range being 70–100%. After day 7: 100% of the estimated need	Day 1: 1/3 of the estimated need Day 2–7: 60% of the estimated need with the acceptable range being 40–60%. After day 7: 100% of the estimated need
Protein provision	1.2–1.5 g/kg /d	1.2–1.5 g/kg /d	1.2–1.5 g/kg /d
Use of prokinetic agent	No	Yes, from Day 1 to Day 7 (metoclopramide or erythromycin)	No
Type of formula	No specific formula is stipulated. Whey protein will be used to supplement protein if the amount of protein in the formula is inadequate		

*EN* enteral nutrition, *BMI* Body Mass Index

#### Calculation of estimated caloric requirement

A simplistic weight-based eq. (25 kcal/kg/d) is used to determine energy requirements [12]. For patients with BMI between 18 and 30 kg/m^2^: daily caloric requirement (kcal) = actual weight (in kilograms) × 25 (kcal). For patients with BMI < 18 kg/m^2^ or BMI > 30 kg/m^2^: daily caloric requirement (kcal) = [height (in cm) – 105] × 25 (kcal).

#### Formulae

The type of enteral feeding formula will be left to discretion of the attending physician as long as it has been approved by China Food and Drug Administration and satisfies the total caloric requirements. No specific formula is stipulated (both specialized and non-specialized formula can be used). Whey protein will be used to supplement protein if the amount of protein in the formula is inadequate.

#### The procedure of enteral feeding

This EN protocol should be initiated within 4 h of randomization. All the participants will receive enteral feeding via nasogastric tubes. After confirmation of the tube tip placement in the stomach, feeding will be started at 30 ml/hour and advanced to 80–100 ml/h within 24 h [22]. Gastric residual volume (GRV) should be checked every 4 h: if GRV < 200 ml, return the aspirate and continue as per the protocol; if GRV > 100 ml, administer the prokinetic drug (metoclopramide or erythromycin); if GRV > 200 ml in two consecutive checks, hold the feeding for at least 4 h, and resume feeding at previous rate when GRV < 200 ml. In all patients receiving EN, the head of the bed should be elevated 30°–45°. EN should not be discontinued automatically for diarrhea. An attempt should be made to evaluate the etiology of diarrhea in order to determine appropriate treatment [12].

#### The timing for initiation of parentral nutrition (PN) in the trial

The exclusive PN will be withheld over the first week after admission if early EN is not feasible. The use of supplemental PN will be considered after 10 days if unable to meet > 60% of energy and protein requirements by the enteral route alone. When clinical signs of intolerance to EN subsides, the provision of PN energy should be discontinued gradually when the patient is able to meet > 60% of target energy requirements by the enteral route [12].

#### Blood glucose management

Blood glucose will be checked every 4 h. The target blood glucose is between 7.7–10.0 mmol/L (140–180 mg/dl) [12]. All the participating centers can use their own standardized protocols to keep the blood glucose in the target range.

### Study procedures

Table 2 shows all the variables required to be measured at every time point of the study. At the time point of baseline screening, demographics, medical history, subtypes of stroke, clinical scores (NIHSS, GCS, Barthel index, NRS 2002), physical examination, and vital signs are recorded. Routine laboratory tests for severe stroke patients are assessed on Day 1 and Day 7, including blood routine, blood electrolyte, transferring, prealbumin, serum lipid, liver and renal function test, electrocardiography, and urine routine. On the day of screening and hospital discharge, comorbidities are recorded. Gastric residual volumes and blood glucose are assessed at 8 am, 12 am, 4 pm, and 8 pm from Day 1 to Day 7. Clinical scores on Day 7, at hospital discharge, and on Day 90 are recorded. Blood pressure, adverse events, and vital signs are monitored during the whole hospitalization, and concomitant treatments are collected.

**Table 2:** Timing and content of study assessments

Items	Day of Enrollment									
	Screening	1	2	3	4	5	6	7	HD	90
Written Informed Consent	●									
Inclusion & exclusion criteria	●									
Demographics	●									
Medical History	●									
BMI & NRS 2002	●									
NIHSS & GCS	●								●	
Barthel Index & mRS	●								●	●
Physical examination	●							●	●	
Laboratory tests		●						●		
Provision of calories and protein		●	●	●	●	●	●	●		
Gastric residual volumes and blood glucose monitoring		●	●	●	●	●	●	●		
Vital signs monitoring	●	●	●	●	●	●	●	●	●	
TSF and MUAC		●						●		
Comorbidities	●								●	
Adverse events		●	●	●	●	●	●	●	●	
Concomitant therapies		●	●	●	●	●	●	●	●	

*HD* hospital discharge, *GCS* Glasgow Coma Scale, *MUAC* mid-upper arm circumference, *NIHSS* National Institute of Health stroke scale, *mRS* modified Rankin Scale, *TSF* triceps skinfold

### Patient safety

Administration of EN is a routine and standardized practice in the neurological intensive care units (N-ICU) and will not cause any special safety issues. The provision of nutrition therapy is based on the latest guidelines from Society of Critical Care Medicine (SCCM) and American Society for Parenteral and Enteral Nutrition (A.S.P.E.N.) [12, 23]. The rest of the medical care for stroke is based on the related guidelines [24–26].

### Outcome measurement

The primary outcome measurement is the three-month functional outcomes evaluated by mRS. A mRS score ≥ 3, major disability or all-cause death, was considered a poor outome. The key secondary outcome is the functional outcome at hospital discharge. Other secondary outcomes include incidence rates of complications during hospitalization (gastric retention, diarrhea, gastrointestinal hemorrhage, and aspiration pneumonia), the disability at hospital discharge (evaluated by GCS, NIHSS, and Barthel Index), and the daily living ability on Day 90 assessed by Barthel Index. Outcomes are evaluated independently by trained researchers who are blind to the clinical data and grouping results. The three-month functional outcomes are assessed via telephone interviews 90 days after the enrollment (within 3 days of this time point is acceptable). If the patient is unable to complete the follow-up interview, the proxy is the spouse or live-in companion.

### Data quality

We carried out four procedures to ensure protocol standardization, control data quality, and minimize bias: (1) Research coordinators from all the participating sites have to attend a training session before the commencement of OPENS study; (2) The Principal Investigator in each research center supervises the conduct of the trial and takes charge of quality control in accordance with the prescribed protocol, applicable regulations and guidelines; (3) Monitoring visits to all the participating sites will be paid via video conferencing after every ten patients are enrolled, to verify eligibility criteria, consent, reported serious adverse events, and anomalous data; (4) The Shaanxi Cerebrovascular Disease Clinical Research Center (SCRC) will release reports on the progress in recruitment, data completeness, and overall research quality every month.

### Data security

Informed consent forms and case report forms will be locked in a cabinet securely with limited access in a secure office. All the digital data will be secured by password and stored in a secure environment protected by firewall. The trial sponsor and principal investigator have access to the final trial dataset.

### Determination of the sample size

The sample size was set at 600 to provide at least 80% power to detect a 15% absolute risk reduction in the primary outcome for patients in the permissive underfeeding group or modified full EN group compared to those in the standard full EN group, using a two-sided significance test with 5% type I error. The following assumptions were made: a primary outcome of 60% in the standard full EN group will be reduced to 45% in the modified full EN group or in the permissive underfeeding group; and there will be 10% non-adherence to the treatment protocol and 3% overall loss to follow-up. The 60% incidence of unfavorable 90-day outcome is derived from one Chinese study on severe stroke (NIHSS > 10) in 2013 [27].

### Statistical and analytical plan

Patients will be analyzed in accordance with the intention to treat principle. Biostatisticians blinded to clinical data in the Shaanxi Cerebrovascular Disease Clinical Research Center will conduct the all the analyses. Baseline characteristics will be processed using univariate analyses. Categorical variables will be summarized as the number and percent, and the continuous variables will be presented as median (interquartile range) or mean (±standard deviation). The proportions of the primary and secondary outcomes among patients randomized to standard full EN group, modified full EN group, and permissive underfeeding group will be compared using the Chi square test. We will calculate the absolute risk reduction, relative risk reduction, and the number needed to treat with modified full EN provision or permissive underfeeding to prevent one death. Logistic multivariate analyses will be conducted to estimate adjusted odds ratios and associated 95% confidential intervals. Two-sided *p* values ≤0.05 will be considered significant. Statistical analysis will be performed with SPSS version 22 software (SPSS Inc., Chicago, IL).

### Protocol amendments

All the protocol amendments have to be agreed upon with sponsor, funding body, and the OPENS Study Group before submission for ethical approval. Subsequent protocol modifications during the trial conduction will be agreed upon with relevant parties such as the trial registry, the trial investigators, and, trial participants, if required.

### Dissemination policy

The results of OPENS trial will be distributed to a wide clinical audience (health professionals, patients, the general public, and policy makers) via publication in an international scientific journal with a high impact factor.

## Discussion

The OPENS trial, the first rigorous trial comparing a modified strategy of full EN (full EN in conjunction with prokinetic drug) and a strategy of permissive underfeeding (40 to 60% of estimated caloric requirements) with a strategy of standard full EN in patients with severe stroke, will provide ClassIevidence in EN feeding for stroke patients with an initial presentation of severe neurological deficits.

Patients with severe stroke often develop malnutrition, which further increases the rate of pneumonia, prolongs hospital length of stay, impedes functional improvement, and contributes to mortality [28]. Therefore, nutrition support is a key component of the critical care of patients with severe stroke, who usually suffer from consciousness impairment, dysphagia, gastroparesis, gastrointestinal bleeding, intestinal barrier dysfunction, and other comorbidities which might cause aspiration, bactrial translocation, sepsis, and malabsorption of food and drug. How to provide enteral nutrition properly and scientifically for patients with severe stroke is a persistent challenge worldwide.

Data concerning the effect of different doses of EN on outcomes in critically ill patients are conflicting. Both hypercaloric and hypocaloric feeding has not been shown to reduce the mortality of critically ill patients [29, 30]. Augmenting energy intake was indicated to increase the time to discharge from intensive care unit (ICU) [31]. Two randomized, controlled trials showed that trophic feeding (up to 500 kcal/d) improved the tolerance of EN [15, 16], but they have been criticized for inadequate provision of protein (0.6–0.8 g/kg/d) [12]. PermiT trial showed that permissive underfeeding strategy (60 to 70% of the estimated caloric requirement in conjunction with full targeted protein intake) was associated with similar outcomes as full EN strategy [17, 18]. As PermiT trial was not designed for patients with severe stroke who had distinct characteristics from patients in general ICU, in this study we aimed to evaluate the effects of permissive underfeeding strategy and standard full EN strategy on clinical outcomes of patients with severe stroke.

Clinical practice guidelines recommended initiating EN within 24–48 h of ICU admission [12]. Unfortunately, about 63% of critically ill patients are intolerant to EN due to gastrointestinal motility dysfunction [32], which is associated with inadequate caloric intake, aspiration, prolonged hospital length of stay, and mortality [33–35]. Metoclopramide and erythromycin are the most commonly used prokinetic agents, and have been demonstrated in placebo-controlled trials to facilitate tolerance to EN in critically ill patients [36, 37]. However, the evidence for the routine addition of prokinetic agents in initial EN for patients with severe stroke is lacking.

The OPENS trial will be the first study to explore the optimum initial feeding strategy for acute severe stroke. There are also some limitations in this study. First, the findings of this study cannot guide enteral feeding in patients with high nutrition risk, because they may need additional parenteral nutrition and they were excluded from this study. Second, blinding of the intervention for researchers was not possible, but all the patients were blind to the grouping results. Third, this study was powered to detect an absolute risk reduction of 15 percentage in three-month poor outcome, thus, a smaller treatment effect cannot be ruled out. Moreover, multivitamin supplementation was not monitored in this study.

In conclusions, we designed a multicenter, randomized, controlled study to investigate whether a strategy of permissive underfeeding and a strategy of full EN in conjunction with prokinetic agents would improve the outcomes in patients with severe stroke. The outputs from this trial will guide the management of initial enteral feeding for patients with severe stroke during the first week of hospitalization.

## Abbreviations

ASPEN: American society for parenteral and enteral nutrition
EN: Enteral feeding
GCS: Glasgow coma scale
GRV: Gastric residual volume
mRS: Modified rankin scale
NIHSS: National institute of health stroke scale
NRS 2002: Nutritional risk screening 2002
OPENS: Optimizing early enteral nutrition in severe stroke
SCCM: Society of critical care medicine
SCRC: The Shaanxi cerebrovascular disease clinical research center
